# The hepatocyte growth factor-expressing character is required for mesenchymal stem cells to protect the lung injured by lipopolysaccharide in vivo

**DOI:** 10.1186/s13287-016-0320-5

**Published:** 2016-04-29

**Authors:** Shuling Hu, Jinze Li, Xiuping Xu, Airan Liu, Hongli He, Jingyuan Xu, Qihong Chen, Songqiao Liu, Ling Liu, Haibo Qiu, Yi Yang

**Affiliations:** Department of Critical Care Medicine, Zhongda Hospital, Southeast University School of Medicine, No.87 Dingjiaqiao Road, Nanjing, 210009 Jiansu P.R. China

**Keywords:** Acute lung injury, Mesenchymal stem cell, Hepatic growth factor, Lung vascular permeability

## Abstract

**Background:**

Acute respiratory distress syndrome (ARDS) is a life-threatening condition in critically ill patients. Recently, we have found that mesenchymal stem cells (MSC) improved the permeability of human lung microvascular endothelial cells by secreting hepatocyte growth factor (HGF) in vitro. However, the properties and functions of MSC may change under complex circumstances in vivo. Here, we sought to determine the role of the HGF-expressing character of MSC in the therapeutic effects of MSC on ARDS in vivo.

**Methods:**

MSC with HGF gene knockdown (MSC-ShHGF) were constructed using lentiviral transduction. The HGF mRNA and protein levels in MSC-ShHGF were detected using quantitative real-time polymerase chain reaction and Western blotting analysis, respectively. HGF levels in the MSC culture medium were measured by enzyme-linked immunosorbent assay (ELISA). Rats with ARDS induced by lipopolysaccharide received MSC infusion via the tail vein. After 1, 6, and 24 h, rats were sacrificed. MSC retention in the lung was assessed by immunohistochemical assay. The lung wet weight to body weight ratio (LWW/BW) and Evans blue dye extravasation were obtained to reflect lung permeability. The VE-cadherin was detected with inmmunofluorescence, and the lung endothelial cell apoptosis was assessed by TUNEL assay. The severity of lung injury was evaluated using histopathology. The cytokines and HGF levels in the lung were measured by ELISA.

**Results:**

MSC-ShHGF with markedly lower HGF expression were successfully constructed. Treatment with MSC or MSC carrying green fluorescent protein (MSC-GFP) maintained HGF expression at relatively high levels in the lung at 24 h. MSC or MSC-GFP decreased the LWW/BW and the Evans Blue Dye extravasation, protected adherens junction VE-cadherin, and reduced the lung endothelial cell apoptosis. Furthermore, MSC or MSC-GFP reduced the inflammation and alleviated lung injury based on histopathology. However, HGF gene knockdown significantly decreased the HGF levels without any changes in the MSC retention in the lung, and diminished the protective effects of MSC on the injured lung, indicating the therapeutic effects of MSC on ARDS were partly associated with the HGF-expressing character of MSC.

**Conclusions:**

MSC restores lung permeability and lung injury in part by maintaining HGF levels in the lung and the HGF-expressing character is required for MSC to protect the injured lung.

**Electronic supplementary material:**

The online version of this article (doi:10.1186/s13287-016-0320-5) contains supplementary material, which is available to authorized users.

## Background

Acute lung injury (ALI) and its severe form, acute respiratory distress syndrome (ARDS), are life-threatening conditions in critically ill patients [1]. Although substantial progress has been made in the treatment of ALI/ARDS, especially in the development of lung-protective ventilation and a fluid-conservation strategy, mortality remains high [2, 3]. Novel therapies for ALI/ARDS are therefore needed.

ALI/ARDS are characterized by diffuse injury to the lung endothelial and epithelial cells, which leads to an increase in alveolar-capillary permeability and alveolar pulmonary edema [4]. The lung endothelium is the first barrier to prevent cells and proteins in the blood from infiltrating into the interstitial space and the alveoli of the lung. Therefore, it is essential to restore injured endothelial cells for the treatment of ALI/ARDS.

Bone marrow-derived mesenchymal stem cells (MSC) are one of several types of stem cells that may have therapeutic effects on damaged organs. MSC are capable of differentiating into different types of cells, secreting paracrine soluble factors, releasing microvesicles (MV), homing to injury sites, and so forth. Many studies show that MSC are beneficial in ALI/ARDS [5–7]. They can restore lung protein permeability, reduce inflammation, and improve survival in lipopolysaccharide- (LPS) or *Escherichia coli*-induced lung injury in mice. However, the underlying mechanism by which MSC restores lung protein permeability in vivo has not yet been fully clarified.

Recently, the strong paracrine property of MSC has been considered to be the principal mechanism for maintaining function in damaged organs [8, 9]. The pulmotrophic factor, hepatocyte growth factor (HGF), which plays an important role in protecting vascular permeability, is a major paracrine soluble factor of MSC. Many studies have detected HGF protein in the cell culture medium of MSC [10, 11]. Furthermore, HGF expression increased when MSC were under hypoxic conditions or stimulated with LPS [10]. In our previous in vitro study [12], we found that, by secreting HGF, MSC improved protein permeability across the human lung microvascular endothelial cell (HMVEC) monolayer and repaired intercellular junctions, including the tight junctions and the adherens junctions of lung endothelial cells. However, in in-vivo conditions, the circumstances for MSC are different due to the unchecked inflammation, severe hypoxemia, and the increased pulmonary alveolar and capillary membrane leakage of ARDS. These all may affect the properties and functions of MSC. Hence, we sought to determine whether the HGF-expressing character of MSC would also be critical for MSC to maintain lung permeability and protect the lung from injury in vivo.

## Methods

### Ethics statement

Male wild-type Sprague-Dawley (SD) rats (Laboratory Animal Center, Shanghai, China) were maintained under specific pathogen-free conditions. The Animal Care and Use Committee of Southeast University approved all experiments involving the use of animals.

### Cell culture

Rat MSC, isolated from the bone marrow of SD rats, and the MSC culture medium were purchased from Cyagen Bioscience, Inc. (Guangzhou, China). 293 T cells were supplied by Cell Bank of Chinese Academy of Sciences. The supplier identified MSC according to cell surface phenotypes and multipotency. Fluorescence-activated cell sorting (FACS) analysis characterized surface phenotypes using the following markers: CD90+, CD44+, CD29+, CD34–, CD45–, and CD11b/c–, and the capacities to differentiate into the adipogenic, osteogenic and chondrogenic lineages were determined by staining with oil red-O, alizarin red or alcian blue, respectively, after culturing in adipogenic, osteogenic or chondrogenic differentiation media (Cyagen Bioscience, Inc., Guangzhou, China) for 2–3 weeks (See Additional file 1).

MSC were cultured in the MSC culture medium made from SD rat MSC basal medium containing 10 % SD rat MSC-qualified fetal bovine serum, 1 % penicillin-streptomycin and 1 % glutamine. The culture medium was changed every 2–3 days and the cells were split when they achieved 90 % confluency. The MSC used for in-vitro studies had been passaged 10 times after lentiviral transduction. MSC with the total passage number <9 were used in the in-vivo experiments. 293 T cells were cultured in Dulbecco’s modified Eagle’s medium (DMEM) containing 10 % fetal bovine serum (Wisent, Inc., St-Bruno, Quebec, Canada), 1 % l-glutamine, and 1 % penicillin-Streptomycin, and incubated at 37 °C in a humidified atmosphere of 5 % CO_2_. Additional materials were provided in Additional file 2. 

### Lentiviral vector-mediated HGF gene knockdown in MSC

MSC with passage number <6 were used for this experiment. Briefly, the rat HGF knockdown constructs expressing short-hairpin RNA targeting endogenous HGF (ShRNA HGF) were encoded into a lentivirus-based ShRNA vector pGLV3/H1/GFP + Puro (LV3) driven by the H1 promoter containing green fluorescent protein (GFP) and puromycin. Target sequences were designed and selected with software Designer 3.0 provided by GenePharma. Additionally, LV3 containing nonspecific ShRNA (LV3-GFP) was used as a negative control. The recombinant vectors were integrated and replicated in *E. coli* Top10 (GenePharma, Shanghai, China).

The recombinant plasmid DNAs were extracted from *E. coli* Top10 and purified using the Plasmid Preparation Kit (GenePharma, Shanghai, China). The purity of the DNA was assessed with a spectrophotometer (Tecan, Switzerland). A260/A280 nm absorbance ratios of 1.8–2.2 suggested a pure DNA sample. Theses plasmids were then separately co-transfected with three packaging plasmids (pGag/Pol, pRev, pVSV-G) into 293 T cells using RNAi-mate (Genepharma, Shanghai, China) according to the manufacturer’s instruction. The lentiviral particles were collected and stored at –80 °C for future use. Titer was obtained by GFP expression assay [13].

MSC were seeded and cultured in six-well plates for 24 h. The lentiviral vectors (carrying LV3-GFP or LV3-GFP ShRNA HGF) were then added to the wells at a multiplicity of infection (MOI) value of 100:1 and cultured with MSC for 24 h. After 24 h, the culture medium was changed, and puromycin was added at the minimal lethal concentration (1.5 μg/ml) for transfected MSC. The puromycin-resistant cells were then collected.

### RNA isolation and quantitative real-time polymerase chain reaction (qRT-PCR)

MSCs treated with LV3-GFP (MSC-GFP) or LV3-GFP-ShRNA HGF (MSC-ShHGF) were collected, respectively. Total RNA was isolated from MSC, MSC-GFP or MSC-ShHGF using TRIzol reagent (Takara Bio, Inc., Kyoto, Japan) according to the manufacturer’s protocol. The quality of the RNA was assessed with a spectrophotometer (Tecan, Switzerland). 260/280 nm absorbance ratios of 1.8–2.2 suggested a pure RNA sample. The RT-PCR primers for rat glyceraldehyde-3-phosphate dehydrogenase (GAPDH) and rat HGF (Table 1) were provided by GenePharma (Shanghai, China). RT-PCR assays were performed following the One-Step RT-PCR protocol described by Funglyn Biotech Inc. (Shanghai, China).

**Table 1:** The primer sequence of genes

Gene	Primer	Primer sequence	PCR amplified products (bp)
GAPDH	Forward primer	5’ > GTGCTGAGTATGTCGTGGAGTCT < 3’	104
	Reverse primer	5’ > GGAAGGGGCGGAGATGA < 3’	
HGF	Forward primer	5’ > GCACCTCCTCCTGCTTCC < 3’	288
	Reverse primer	5’ > CCAAACCCTTTTTTCACTCCA < 3’	

*bp* base pair, *GAPDH* glyceraldehyde-3-phosphate dehydrogenase, *HGF* hepatocyte growth factor, *PCR* polymerase chain reaction

### Western blotting analysis

MSC, MSC-GFP, and MSC-ShHGF were collected after transduction with lentiviral vector. Total cellular protein from either MSC, MSC-GFP, or MSC-ShHGF was extracted and separated using SDS-PAGE gels (10 %), as previously described [14]. Protein was then incubated with primary antibodies to HGF (1:600 dilution; Santa Cruz Biotechnology, Inc., Santa Cruz, CA, USA) or β-actin (1:10,000 dilution; Abcam Ltd., Cambridge, UK). The blots were washed three times and then incubated with goat anti-rabbit IgG conjugated with horseradish peroxidase (HRP; Zhongshan Golden Bridge Biotechnology Co., Ltd, China). Immunoreactive complexes were visualized using chemiluminescence reagents (Thermo Scientific).

### Evaluation of HGF levels by ELISA

MSC, MSC-GFP, and MSC-ShHGF were seeded in a 12-well plate at a density of 1 × 10^5^ cells per well. After 12 h the culture medium was changed, and MSC were cultured in an incubator at 37 °C, 5 % CO_2_ for 24 h. The culture medium was then collected and HGF protein levels in the culture medium were quantified using an enzyme-linked immunosorbent assay (ELISA) kit (ExCellBio, Shanghai, China) according to the manufacturer’s instructions.

### LPS-induced ALI in rats

To induce ALI, 6- to 8-week-old wild-type SD rats received an intratracheal instillation of LPS (2 mg/kg, *E. coli* 0111:B4; Sigma-Aldrich, St. Louis, MO, USA) dissolved in 100 μl phosphate-buffered saline (PBS; Wisent, Inc., St-Bruno, Quebec, Canada) as described previously [15]. PBS, MSC, MSC-GFP, or MSC-ShHGF (5 × 10^6^ cells resuspended in 100 μl PBS) were injected into the tail vein 5 h after LPS challenge. Rats without LPS challenge were injected with PBS as a control. Rats were sacrificed at 1, 6 and 24 h after MSC injection, and the lung lobes were collected for further analysis.

### Measurement of lung edema

Lung wet weight to body weight (LWW/BW) ratios, which reflected the severity of lung vascular permeability and lung edema, were obtained from the control, ALI, MSC, MSC-GFP, and MSC-ShHGF group.

### Evans blue dye leakage

For the control, ALI, MSC-GFP, and MSC-ShHGF group (n = 6 per group, see Additional file 3 for sample size calculation), Evans blue dye (20 mg/kg in 1 ml saline; Sigma-Aldrich, St. Louis, MO, USA) was injected into the tail vein of the rats. After 30 min, the right ventricle of the heart was perfused with 100 ml heparinized saline to clean up the dye remaining in the lung vascular system. When all the dye had been cleared from the lung vascular system, the whole lung was collected. Lung tissue (100 mg) from the right lobe was then incubated in formamide (Sigma-Aldrich, St. Louis, MO, USA) for 24 h at 60 °C, and the concentration of Evans blue dye was measured using a spectrophotometer at 630 nm (Tecan, Switzerland).

### Immunohistochemical staining

Immunohistochemical analysis was performed to determined expression of GFP in the lung. Sections (5 μm thick) were cut from paraffin-embedded tissues. After being routinely deparaffinized and dehydrated, sections were subjected to antigen retrieval by microwave treatment in boiling 0.01 M citrate buffer (pH 6.0) for 20 min. Then the sections were blocked in 1 % bovine serum albumin (BSA) for 1 h at 37 °C and incubated with anti-GFP primary antibody (Abcam Ltd., Cambridge, UK) overnight at 4 °C, followed by HRP conjugated goat anti-rabbit IgG (Santa Cruz Biotechnology, Inc., Santa Cruz, CA, USA). After washing with 0.1 M PBS, the sections were reacted with a staining solution containing 0.03 % 3,3’-diaminobenzidine tetrahydrochloride (Nanjing Lufei Biotechnology Co., Ltd., Nanjing, China) for 5–10 min at room temperature. Mayer’s hematoxylin was used for counterstaining. The sections were examined with an Olympus IX71 microscope (Olympus Co., Tokyo, Japan). Cells with GFP were counted by a pathologist based on five randomly selected high-power fields (×400).

### Immunofluorescence

To measure co-localization of VE-cadherin in the lung, rat lungs were collected gently and then fixed in 4 % paraformaldehyde at 4 °C. Twenty four hours later, lung tissue was frozen in optimal cutting temperature medium (OCT; Sakura Finetek USA, Inc., Torrance, CA, USA) and cut into 5-μm-thick sections. Then the sections were stained with anti-VE-cadherin antibody (Santa Cruz Biotechnology, Inc., Santa Cruz, CA, USA), followed by Fluorescence (FITC)-AffiniPure Donkey Anti-Rabbit IgG (H + L; Jackson ImmunoResearch Inc., PA, USA), and mounted with 4,6-diamidino-2-phenylindole (DAPI; Sigma-Aldrich). Fluorescence was monitored with an Olympus IX71 microscope (Olympus Co., Tokyo, Japan). The mean fluorescence intensity of VE-cadherin of five randomly chosen high-power fields per lung lobe section per rat was assessed and calculated.

### TUNEL assay

To assess the apoptosis of endothelial cells in the lung, the right lobe was collected and fixed in 4 % paraformaldehyde at 4 °C for 24 hours, and the lung sections were stained with a TUNEL Apoptosis Assay kit (Nanjing Lufei Biotechnology Co., Ltd., Nanjing, China). The sections were observed with an Olympus IX71 microscope (Olympus Co., Tokyo, Japan). Endothelial cells were quantified by a pathologist based on five randomly selected high-power fields (×400). The number of apoptotic lung endothelial cells and total lung endothelial cells were recorded, and the apoptosis index of lung endothelial cells, which was defined as the number of apoptotic lung endothelial cells divided by the total number of lung endothelial cells, was used to assess the severity of lung endothelial cell apoptosis.

### Lung histopathology

To examine the severity of lung injury, the right lobes were collected at 1, 6 and 24 h after MSC infusion and then fixed in 4 % paraformaldehyde. After fixation, the lungs were embedded in paraffin and cut into 5-μm sections. The lung sections were stained using a hematoxylin and eosin staining kit purchased from the Beyotime Institute of Biotechnology. The severity of lung injury was quantified and assessed using a total lung injury score as we previously described [15].

### HGF expression in the lung

To detect the HGF levels in the lung, the left lung was collected and stored at –80 °C. The lung tissue was homogenized thoroughly in PBS solution and then centrifuged at 3000 rpm for 20 min at 4 °C. The levels of HGF in the supernatant were then measured using an ELISA kit (ExCellBio, Shanghai, China), according to the manufacturer’s instructions.

### Measurement of cytokines in the lung

The left lobe was snap-frozen and later processed for lung homogenization. Then the lung homogenate was centrifuged at 3000 rpm for 20 min at 4 °C and the supernatant was collected. The concentrations of interleukin (IL)-1β and IL-10 in the supernatant were evaluated using ELISA kits (NeoBiosicence, Shenzhen, China), according to the manufacturer’s instructions.

### Statistical analysis

Data from in vitro experiments are shown as the mean ± standard deviation. Data from in vivo experiments are shown as individual point + median. Statistical analyses were performed using SPSS 16.0. For comparisons between multiple groups, one-way analysis of variance (ANOVA) followed by Bonferroni’s post-hoc test was used. *p* values <0.05 were considered to be statistically significant.

## Results

### The efficiency of lentiviral vector-mediated HGF gene knockdown

**Fig. 1:**
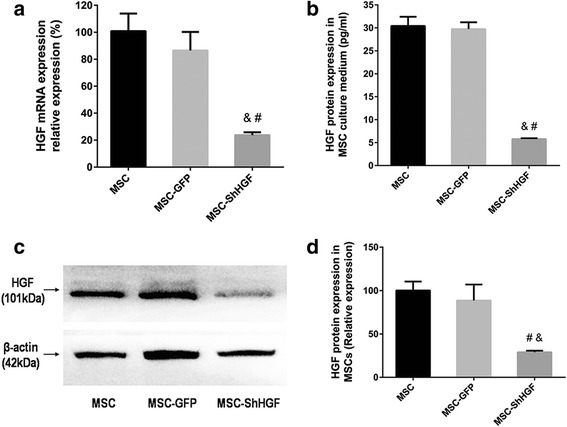
Measurement of hepatocyte growth factor (*HGF*) expression in mesenchymal stem cells (*MSC*) after HGF gene knockdown. **a** Evaluation of HGF mRNA expression in the MSC, MSC-GFP, and MSC-ShHGF groups. **b** Detection of HGF protein levels in the culture medium of the MSC, MSC-GFP, and MSC-ShHGF groups. **c** Image for quantification of HGF protein expression in the MSC, MSC-GFP, and MSC-ShHGF groups by Western blotting analysis. **d** The quantitative results for Western blotting analysis. *n* = 3. ^&^ *p* < 0.05 vs. the MSC group; ^#^ *p* < 0.05 vs. the MSC-GFP group. *GFP* green fluorescent protein, *ShHGF* hepatocyte growth factor gene knockdown

HGF mRNA levels were detected by qRT-PCR. The result showed that HGF mRNA expression was significantly lower in the MSC-ShHGF group than in the MSC (*p* < 0.05) and MSC-GFP group (*p* < 0.05). However, there was no significant difference between the MSC and MSC-GFP group (*p* > 0.05) (Fig. 1a) in the HGF mRNA expression. The result from Western blotting analysis (Fig. 1c and d) showed that the HGF protein expression in the cytoplasm was decreased in the MSC-ShHGF group compared with the MSC (*p* < 0.05) and MSC-GFP group (*p* < 0.05). The HGF protein levels in the cell culture medium of the MSC-ShHGF group were also significantly lower those that in the MSC (*p* < 0.05) and MSC-GFP groups (*p* < 0.05) (Fig. 1b). However, there was no significant difference in HGF protein levels between the MSC and MSC-GFP group in either the cytoplasm or the cell culture medium. Taken together, these results suggested that lentiviral-mediated HGF gene knockdown was efficient.

### MSC retention in the lung at 24 h

**Fig. 2:**
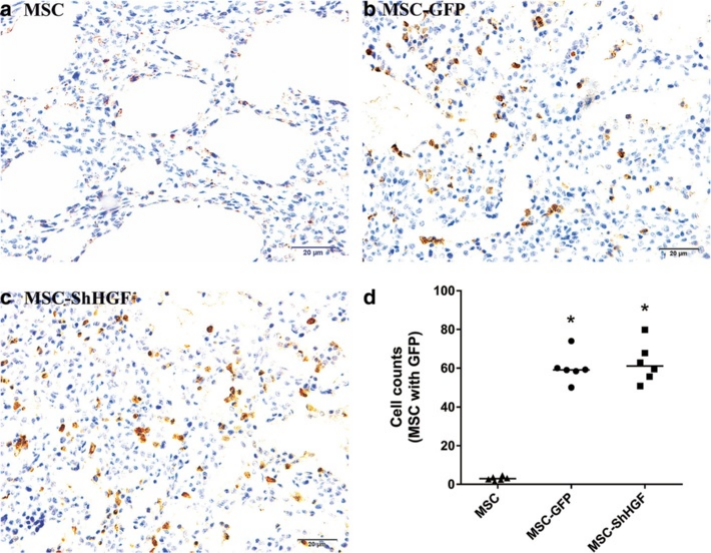
Mesenchymal stem cell (*MSC*) retention in the lung at 24 h after MSC infusion. **a,** **b**, **c** Representative images of MSC retention in the lung in different groups at 24 h after MSC infusion (×400). **d** The quantitative result of MSC retention in the lung in different groups at 24 h after MSC injection. *n* = 6. *p < 0.05 vs. the MSC group. *GFP* green fluorescent protein, *ShHGF* hepatocyte growth factor gene knockdown

To measure the MSC retention in the lung tissue after cell infusion, immunohistochemical assays were performed for the GFP carried by MSC. As Fig. 2 shows, no significant differences were found in the retention of MSC with GFP in the lung tissue between the MSC-GFP and MSC-ShHGF groups (*p* > 0.05), demonstrating that HGF gene knockdown in MSC had no significant impact on MSC homing to the lung in respond to lung injury.

### HGF levels in the lung after MSC treatment

**Fig. 3:**
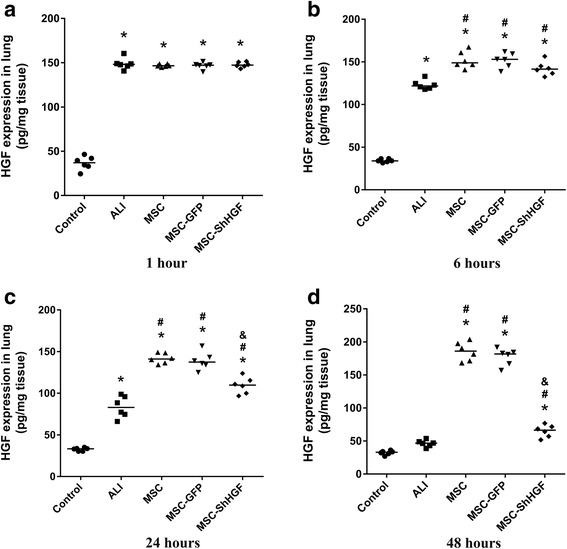
Hepatocyte growth factor (*HGF*) expression in injured lung after mesenchymal stem cell (*MSC*), MSC-GFP, or MSC-ShHGF treatment. HGF protein levels in the lung tissue of different groups at **a** 1, **b** 6, **c** 24, and **d** 48 h after MSC delivery were measured by ELISA. *n* = 6. *p < 0.05 vs. the control group; ^#^ *p* < 0.05 vs. the ALI group; ^&^ *p* < 0.05 vs. the MSC group. *GFP* green fluorescent protein, *ShHGF* hepatocyte growth factor gene knockdown

To examine the effect of MSC infusion on HGF levels in ALI rats, we measured the HGF concentration in the lung. As Fig. 3 shows, HGF protein levels in the lung increased at 1 h after LPS challenge and then gradually decreased at 6, 24 and 48 h (*p* < 0.05). MSC treatment significantly increased HGF levels at 6, 24 and 48 h (*p* < 0.05). When the HGF gene was knocked down in MSC, the HGF level in the lung decreased significantly compared with the MSC or MSC-GFP group at 24 and 48 h (*p* < 0.05). Since no differences were found in the retention of MSC with GFP in the lung tissue between the MSC-GFP and MSC-ShHGF groups, these results suggest that MSC regulated HGF level in the injured lung in part through secreting HGF. Additional results were provided in Additional file 2.

### The effect of MSC on pulmonary vascular permeability

**Fig. 4:**
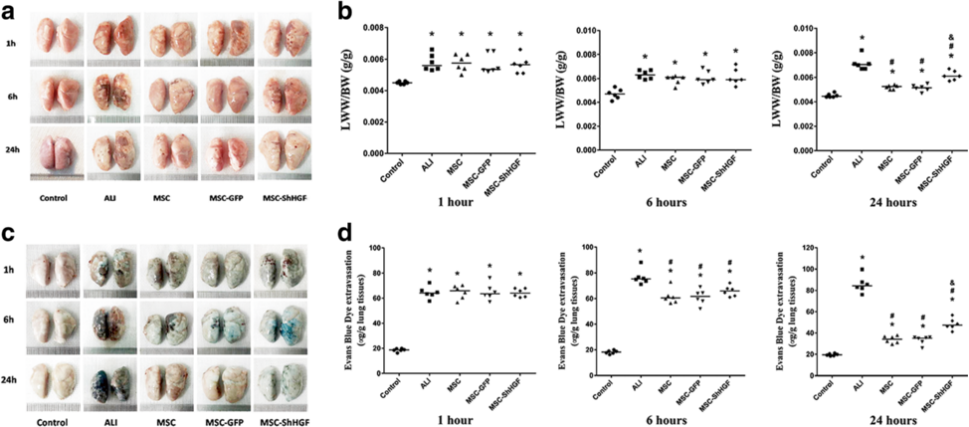
The effect of mesenchymal stem cells (*MSC*), MSC-GFP, and MSC-ShHGF on lung vascular permeability. **a** Representative lung images in different groups at 1, 6 and 24 h after MSC treatment. **b** Comparison of the lung wet weight to body weight ratio (*LWW/BW*) in different groups at 1, 6 and 24 h after MSC injection. **c** Representative images of Evans blue dye leakage from vascular to lung tissues in different groups 1, 6 and 24 h after delivery of MSC. **d** The quantitative result of Evans blue dye leakage. *n* = 6. *p < 0.05 vs. the control group; ^#^ *p* < 0.05 vs. the acute lung injury (*ALI*) group; ^&^ *p* < 0.05 vs. the MSC group. *GFP* green fluorescent protein, *ShHGF* hepatocyte growth factor gene knockdown

In this study, LWW/BW and Evans blue dye extravasation were used to evaluate the role of the HGF-expressing character of MSC in the therapeutic effect of MSC on lung vascular permeability. The results (Fig. 4a and b) showed that LPS stimulation increased LWW/BW markedly at 1, 6 and 24 h (*p* < 0.05). After treatment with MSC, MSC-GFP, or MSC-ShHGF, the LWW/BW was decreased significantly at 24 h in the ALI rats (*p* < 0.05). More interestingly, the LWW/BW in rats treated with MSC with HGF gene knockdown was significantly higher than that in rats treated with MSC or MSC-GFP (*p* < 0.05). Furthermore, as Fig. 4c and d show, LPS stimulation also increased Evans blue dye extravasation from the lung vascular to the lung interstitial space and lung alveoli at 1, 6 and 24 h (*p* < 0.05). With MSC, MSC-GFP, or MSC-ShHGF treatment, the Evans blue dye extravasation decreased markedly at 6 and 24 h (*p* < 0.05) but, in the MSC-ShHGF group, the Evans blue dye extravasation was significantly higher compared with the MSC or MSC-GFP group (*p* < 0.05). These results indicate that MSC improved vascular permeability in lung tissue injured by LPS, and that the HGF-expressing character of MSC was required for MSC to restore this lung vascular permeability.

### VE-cadherin expression in the lung following MSC injection

**Fig. 5:**
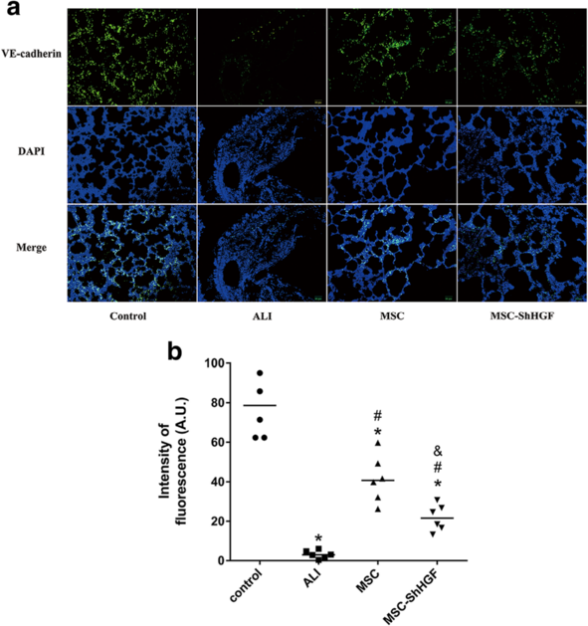
Detection of the changes of VE-cadherin in the lung tissue after mesenchymal stem cell (*MSC*) injection by immunofluorescence. **a** Intercellular junctions were evaluated by detection of the adherens junction protein VE-cadherin using fluorescence microscopy 24 h after MSC-ShHGF treatment (×200; *blue*, DAPI; *green*, VE-cadherin). **b** The quantitative result of VE-cadherin expression in the lung in different groups at 24 h after MSC injection. *n* = 6. *p < 0.05 vs. the control group; ^#^ *p* < 0.05 vs. the ALI group; ^&^ *p* < 0.05 vs. the MSC group. *ALI* acute lung injury, *AU* arbitrary units, *DAPI* 4,6-diamidino-2-phenylindole, *ShHGF* hepatocyte growth factor gene knockdown

To investigate the effect of MSC with HGF gene knockdown on intercellular junctions in the lungs of rats with ALI, we observed the expression of the adherens junction protein VE-cadherin in the rat lung by immunofluorescence at 24 h. As Fig. 5 shows, the VE-cadherin expression in lung tissue was decreased markedly after intratracheal instillation of LPS at 24 h (*p* < 0.05). After treatment with MSC or MSC with HGF gene knockdown, the VE-cadherin expression was increased in the lung compared with the ALI group (*p* < 0.05). However, the increase in VE-cadherin expression in the MSC-ShHGF group was markedly lower than that in the MSC group (*p* < 0.05). These results suggest that MSC had a protective effect on the adherens junction of cells in the lung, and that this protective effect required the HGF expressed by MSC.

### Assessment of endothelial cell apoptosis in the injured lung after MSC treatment by TUNEL assay

**Fig. 6:**
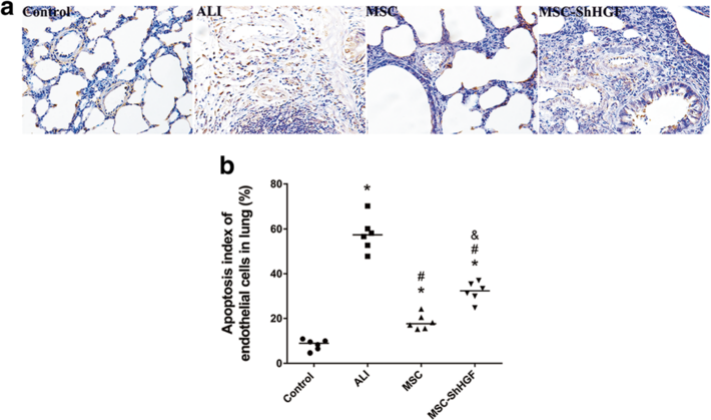
The evaluation of vascular endothelial cell apoptosis in the lung. **a** Representative images of lung endothelial cell apoptosis in different groups 24 h after mesenchymal stem cell (*MSC*) treatment (×400). **b** The quantitative result of the apoptosis index of lung endothelial cells in different groups at 24 h after MSC injection. *n* = 6. *p < 0.05 vs. the control group; ^#^ *p* < 0.05 vs. the ALI group; ^&^ *p* < 0.05 vs. the MSC group. *ALI* acute lung injury, *ShHGF* hepatocyte growth factor gene knockdown

To evaluate the effect of the HGF-expressing character of MSC on endothelial apoptosis in the ALI lung, we carried out a TUNEL assay 24 h after MSC treatment. As Fig. 6 shows, the apoptosis index of the lung endothelium was increased after LPS challenge, whereas it decreased to a large degree after MSC treatment. However, in MSC with gene knockdown, the apoptosis index of the lung endothelium was significantly higher than that in the MSC or the MSC-GFP group. These results indicate that MSC treatment was able to protect lung endothelial cells from apoptosis in the lung of rats with ALI, and that this protective effect was related to the ability of MSC to release the HGF protein.

### Histological evaluation of the therapeutic potential of MSC in ALI rats

**Fig. 7:**
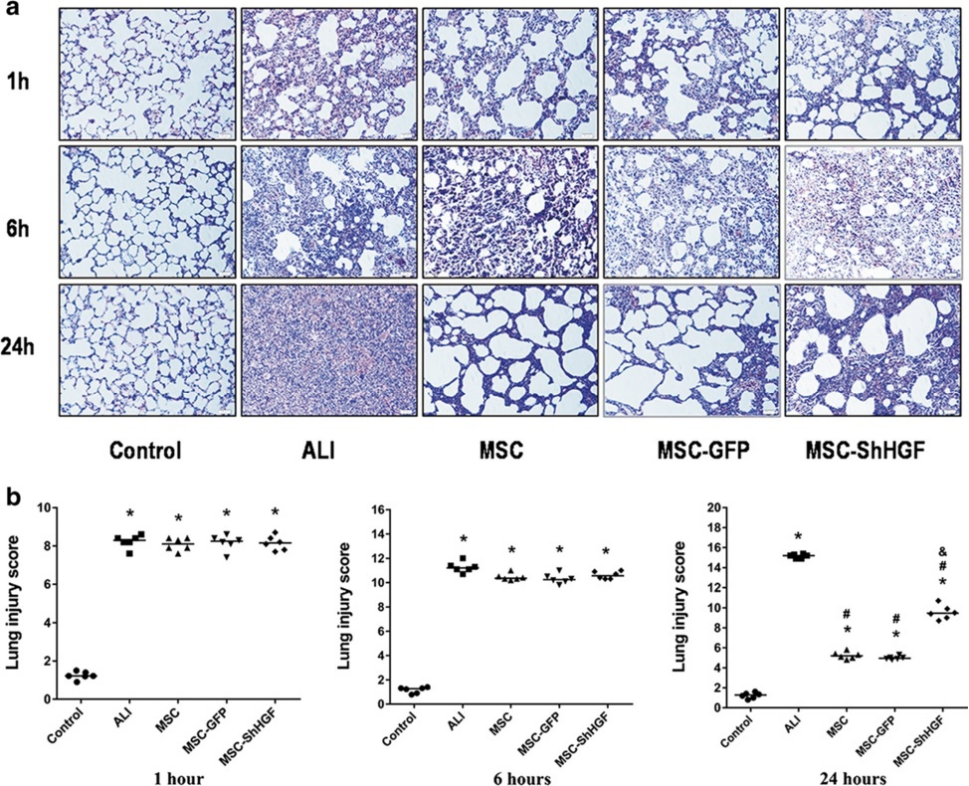
Histological evaluation of the therapeutic potential of mesenchymal stem cells (*MSC*), MSC-GFP, or MSC-ShHGF in acute lung injury (*ALI*) rats. **a** Hematocylin and eosin staining images (×100) of lung sections in each group at 1, 6 and 24 h. **b** Quantitative analysis of the lung injury scores in each group at different time points. *n* = 6. *p < 0.05 vs. the control group; ^#^ *p* < 0.05 vs. the ALI group; ^&^ *p* < 0.05 vs. the MSC group. *GFP* green fluorescent protein, *ShHGF* hepatocyte growth factor gene knockdown

As Fig. 7 shows, histopathology indicated extensive inflammatory infiltrates, marked inter-alveolar septal thickening, and diffuse interstitial and alveolar edema at 1, 6 and 24 h in the ALI group. The lung injury score was 8.23 at 1 h, 10.6 at 6 h and 15.15 at 24 h in the ALI group. The administration of MSC, MSC-GFP, and MSC-ShHGF significantly attenuated lung injury at 24 h (*p* < 0.05). However, the lung injury score in the MSC-ShHGF group (9.53) was significantly higher than that in the MSC (5.23) and MSC-GFP group (4.98) (*p* < 0.05).There was no significant difference in lung injury score between the MSC and MSC-GFP group. These results suggest that MSC had a protective effect on lung injury, and that this effect was diminished when the HGF gene was knocked down in MSC.

### The effect of MSC on cytokine levels in the injured lung after LPS-induced ALI in rats

**Fig. 8:**
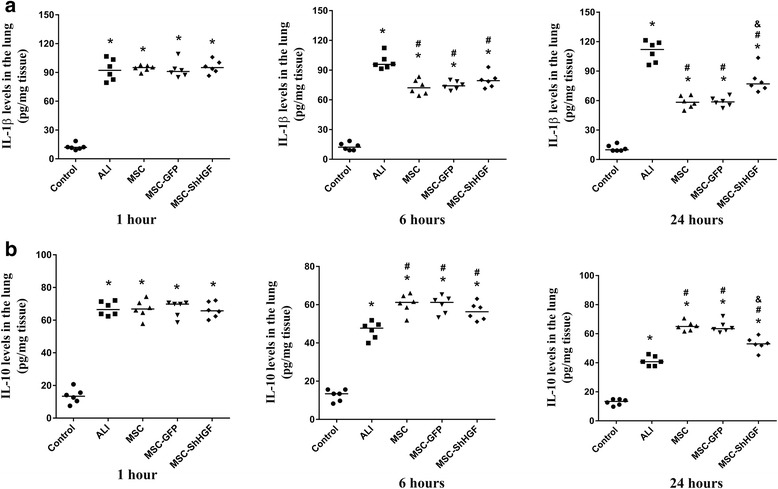
The effect of mesenchymal stem cell (*MSC*), MSC-GFP, and MSC-ShHGF on cytokine levels in the lung. **a** IL-1β levels in lung tissues in different groups at 1, 6 and 24 h after MSC treatment measured by ELISA. **b** IL-10 levels in lung tissues in different groups at 1, 6 and 24 h after MSC treatment measured by ELISA. *n* = 6. *p < 0.05 vs. the control group; ^#^ *p* < 0.05 vs. the ALI group; ^&^ *p* < 0.05 vs. the MSC group. *ALI* acute lung injury, *GFP* green fluorescent protein, *IL* interleukin, *ShHGF* hepatocyte growth factor gene knockdown

In this study, we also investigated the effect of MSC-GFP and MSC with HGF gene knockdown on IL-10 and IL-1β production in the lung. As Fig. 8a shows, the IL-1β level in the lung was markedly increased after LPS challenge at 1, 6 and 24 h (*p* < 0.05). After treatment with MSC, MSC-GFP, and MSC-ShHGF, IL-1β levels in the lung were significantly decreased at 6 and 24 h (*p* < 0.05). There was no significant difference in the lung IL-1β levels between the MSC-ShHGF, MSC, and MSC-GFP group at 6 h. However, there was a significant difference between the MSC-ShHGF and the MSC or MSC-GFP group at 24 h (*p* < 0.05).

The IL-10 level in the lung was significantly decreased after LPS stimulation at 1, 6 and 24 h (*p* < 0.05) (Fig. 8b). However, it was markedly increased after MSC, MSC-GFP, or MSC-ShHGF treatment at 6 and 24 h. In addition, compared with the MSC or MSC-GFP group, the IL-10 level in the lung were significantly lower in the MSC-ShHGF group. These results indicate that MSC reduced LPS-induced inflammation in the lung, and that the HGF-expressing characteristic of MSC played an important part in this effect.

## Discussions

Our previous in vitro studies showed that, by secreting the protective soluble factor HGF, MSC are able to restore the permeability of the HMVEC monolayer following LPS-induced injury [12]. By producing HGF, MSC may maintain the integrity of the injured endothelial monolayer by restoring endothelial intercellular junctions, decreasing caveolin-1 protein expression, and inducing proliferation in endothelial cells. Here, we knocked down HGF in MSC to examine the role of the HGF-expressing character of MSC in the therapeutic effect of MSC on lung permeability and lung injury in vivo.

Firstly, we successfully constructed a stable and long-term MSC cell line with low HGF expression by employing a lentiviral vector-mediated HGF gene knockdown technique. Our data showed that MSC-ShHGF at the tenth passage had notably low HGF mRNA and protein expression. Moreover, HGF levels in the cell culture medium of MSC-ShHGF also markedly decreased. The successful construction of MSC-ShHGF facilitates our further investigations into the role of the HGF-expressing character of MSC in the protective effect of MSC on lung endothelial permeability and lung injury in vivo.

According to our data from the in vivo study, the HGF level in the lung was markedly elevated after LPS challenge. However, it began to decrease at 6 h and was further reduced to approximately half by 24 h. After MSC treatment, the HGF level increased significantly. Interestingly, when the HGF gene was knocked down in MSC, the HGF protein levels in the injured lung were significantly decreased at 24 h but were still higher than those in the ALI group. However, there was no significant difference in MSC retention in the injured lung between the MSC-GFP and MSC-ShHGF groups as shown by the immunohistochemical assay. Taken together, these results suggested that the HGF-expressing character of MSC was required for MSC to increase the HGF levels in the lung, which then exerted a protective effect on the injured lung. There are studies showing that the intravenous or the intra-organ administration of HGF reduced organ injury [12, 16–18]. Recently, studies [19–21] have also demonstrated that MSC overexpressing HGF elevated the HGF level in injured organs and delivered a stronger therapeutic effect compared with normal MSC. Here, our results are consistent with these previous findings.

In this study, data from the LWW/BW and Evans blue dye extravasation assay showed that the HGF-expressing character of MSC played a modest role in the protective effect of MSC on lung endothelial permeability. HGF-expressing MSC have a beneficial effect on maintaining the integrity of the lung endothelium. This may be related to the ability of HGF itself to protect intercellular junctions, as our data demonstrated, preventing endothelial cell apoptosis. Previous studies have demonstrated that HGF had a strong angiogenic effect [22–24]. This could facilitate endothelial cell proliferation [25], prevent apoptosis [26, 27], and restore intercellular junctions and cytoskeleton structures [28, 29] in vitro. HGF administration could also improve the prognosis of lung injury caused by inflammation, oxidative stress, radiation, fibrosis, and so forth [17, 30, 31], which may be partly related to the effect of HGF on repairing the lung endothelium.

In addition to restoring lung endothelial permeability, the HGF-expressing character also plays a positive role in the beneficial effect of MSC on lung inflammation induced by LPS. As shown in Fig. 8, IL-1β levels were significantly higher and IL-10 levels were lower in the MSC-ShHGF group compared with the MSC or MSC-GFP group. This finding suggested that the ability of MSC to control lung inflammation was diminished after the HGF gene in MSC was knocked down. Possible explanations for this result include: 1) HGF has a direct anti-inflammatory effect as demonstrated by previous studies [32, 33]; or 2) the decreased infiltration of immunocytes from the blood to the interstitial space and alveoli of the lung due to the improvement of endothelial permeability related to HGF.

Finally, in the current study, we also assessed lung injury using histopathology and lung injury score (Fig. 7). The improvements in histopathology and lung injury score typically occurred 24 h after MSC treatment. However, in the MSC-ShHGF group this improvement was relatively small compared with the MSC or MSC-GFP group. These results suggested that HGF secretion was required for MSC to exert a better therapeutic effect in ALI. Recently, several studies demonstrated that HGF gene modification (overexpression) in MSC could enhance the therapeutic effect of MSC on injured organs such as the heart [34], liver [35], intestine [33], and lung [30]. Here, we inhibited HGF expression in MSC with a method using lentiviral vector and found out that the therapeutic effects of MSC on lung injury were diminished to some degree. This result is consistent with the previous studies and indicates a modest role of the HGF-expressing character of MSC in the therapeutic effects of MSC on ALI. In summary, our results showed that the HGF-expressing property of MSC was required for MSC-based therapies in lung injury.

There are some limitations to this study. Firstly, adherens junctions were the only type of intercellular junction we examined in this study. Therefore, further studies are needed to clarify the effect of HGF protein production in MSC on the endothelial tight junctions and cytoskeleton in rats with ALI. Secondly, we did not investigate the cellular origin of HGF in the lung, which may help us to understand the underlying mechanism of the beneficial effect of the HGF-expressing character of MSC in ALI more clearly. Moreover, we did not explore the effect of MSC with HGF overexpression on ALI. This would also provide some additional evidence for this work. Finally, we only focused on the short-term effect (24 h) of MSC-ShHGF on ALI. The long-term effects remain unknown and merit further investigation.

## Conclusion

In conclusion, the results presented here suggest that MSC restores lung vascular permeability, which might be associated with the protective effects of MSC on the adherens junction protein VE-cadherin and lung vascular endothelial cell apoptosis, reducing inflammation, and attenuating lung injury in LPS-induced ALI in rats in part by maintaining the HGF level in the injured lung. Moreover, the HGF-expressing character is required for MSC to protect the injured lung.

## Additional files

Additional file 1: Identification of MSC isolated from the bone marrow of SD rats. (DOCX 21.7 mb) Additional file 2: Additional materials and results. (DOCX 8.71 mb) Additional file 3: Equation for samlpe size calculation. (DOCX 13.5 kb)

## Abbreviations

ALI: acute lung injury
ARDS: acute respiratory distress syndrome
ELISA: enzyme-linked immunosorbent assay
GFP: green fluorescent protein
HGF: hepatocyte growth factor
HMVEC: human lung microvascular endothelial cell
HRP: horseradish peroxidase
IL: interleukin
LPS: lipopolysaccharide
LV3-GFP: lentivirus carrying GFP
LV3-ShRNA HGF: lentivirus carrying green fluorescent protein and hepatocyte growth factor gene knockdown
LWW/BW: lung wet weight to body weight ratio
MSC: mesenchymal stem cells
MSC-GFP: mesenchymal stem cells carrying green fluorescent protein
MSC-ShHGF: mesenchymal stem cells with hepatocyte growth factor gene knockdown
PBS: phosphate-buffered saline
qRT-PCR: quantitative real-time polymerase chain reaction
SD: Sprague-Dawley
Sh: short-hairpin
