# Supplementary material for: The hepatocyte growth factor-expressing character is required for mesenchymal stem cells to protect the lung injured by lipopolysaccharide in vivo
Source: Stem Cell Res Ther. 2016 Apr 29;7:66. doi: 10.1186/s13287-016-0320-5 (PMC4850641; doi:10.1186/s13287-016-0320-5)
Supplement: Additional file 1: — Identification of MSC isolated from the bone marrow of SD rats. (DOCX 21.7 mb) [file 13287_2016_320_MOESM1_ESM.docx]

**

**

**Figure S1 Identification of MSC isolated from the bone marrow of SD rats (Data were provided by Cyagen Bioscience, Inc., Guangzhou, China).** (A) MSC cell surface markers, including CD90+, CD44+, CD34+, CD29-, CD45-, and CD11b/c-were determined by flow cytometry. (B) The quantitative flow cytometry analysis of surface markers expression on MSC. (C) The morphology of MSC at the 6^th^ passage (×100) and the multilineage differentiation capacities of MSC, including adipogenic differentiation staining with oil red-O (D, ×200), osteogenic differentiation staining with alizarin red (E, ×200) and chondrogenic differentiation staining with alcian Blue (F, ×200) were observed with a microscope.
