# Supplementary material for: The hepatocyte growth factor-expressing character is required for mesenchymal stem cells to protect the lung injured by lipopolysaccharide in vivo
Source: Stem Cell Res Ther. 2016 Apr 29;7:66. doi: 10.1186/s13287-016-0320-5 (PMC4850641; doi:10.1186/s13287-016-0320-5)
Supplement: Additional file 2: — Additional materials and results. (DOCX 8.71 mb) [file 13287_2016_320_MOESM2_ESM.docx]

**Methods**

**Normal human lung fibroblasts (NHLF) and Cell culture**

Normal human lung fibroblasts cell lines and the NHLF cell culture medium consisting of Supplements and Growth Factors (FGM^TM^-2 SingleQuots^TM^) were purchased from LONZA (Walkersville, Maryland, USA). Briefly, NHLF were cultured in the NHLF culture medium with 1% Penicillin-Streptomycin and incubated at 37℃ in a humidified atomosphere of 5% CO_2_. The culture medium was changed every 2-3 days and the cells were split when they were 70-80% confluent.

**LPS-induced ALI in rats**

To induce ALI, six to eight-week-old wild-type SD rats received an intra-tracheal instillation of LPS (2 mg/kg, Escherichia coli 0111:B4; Sigma-Aldrich, St. Louis, MO, USA) dissolved in 100 µl phosphate buffered saline (PBS, Wisent, Inc., St-Bruno, Quebec, Canada). PBS, NHLF, MSC or MSC-ShHGF (5×10^6^ cells re-suspended in 100 µl PBS) was injected into the tail vein five hours after LPS challenge. Rats without LPS challenge were injected with PBS as a control. Rats were sacrificed at 24 hours after cells injection. Wet lung weight and body weight of rats in each group were obtained, and the lung lobes were collected for further analysis.

**Results**

**
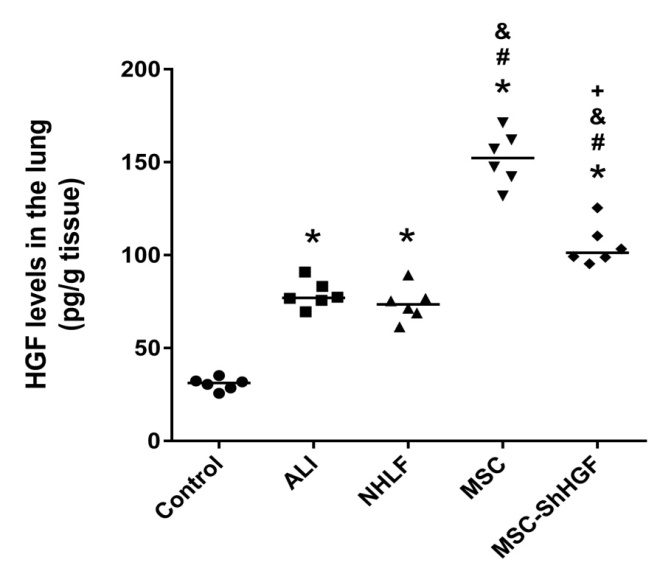
**

**Figure S2. HGF expression in the injured lung after NHLF, MSC or MSC-ShHGF injection at 24 hours.** HGF levels in lung tissue of each group at 24 hours after cells delivery measured by ELISA. The lung HGF levels increased significantly after LPS induced injury. HGF levels in MSC treated group were statistically higher than that in the ALI or NHLF group at 24 hours. However, HGF levels in the lung tissue were decreased dramatically when HGF gene was knockdown in the MSC but still higher than that in the ALI or NHLF group at 24 hours. There was no significant difference in the lung HGF levels between the ALI and NHLF groups. (n=6; ^＊^*p*<0.05 vs. the control group, ^＃^*p*<0.05 vs. the ALI group, ^&^*p*<0.05 vs. the NHLF group, , ^+^*p*<0.05 vs. the MSC group).


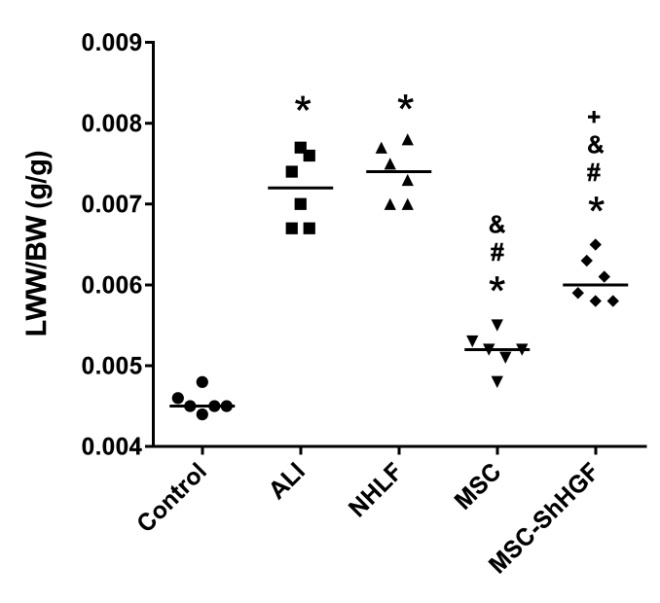


**Figure S3.The effect of MSC or MSC-ShHGF on the wet lung-to-body weight ratio (LWW/BW) at 24 hours.** Comparisons of LWW/BW in different groups at 24 hours after cells treatment. LWW/BW was increased dramatically at 24 hours after LPS instillation. With MSC treatment, LWW/BW was statistically decreased compared with the ALI or NHLF group. However, it was increased significantly when HGF gene was knockdown in MSC. There was no significant difference in the LWW/BW between the ALI and NHLF groups. (n=6; ^＊^*p*<0.05 vs. the control group, ^＃^*p*<0.05 vs. the ALI group, ^&^*p*<0.05 vs. the NHLF group, ^+^*p*<0.05 vs. the MSC group).


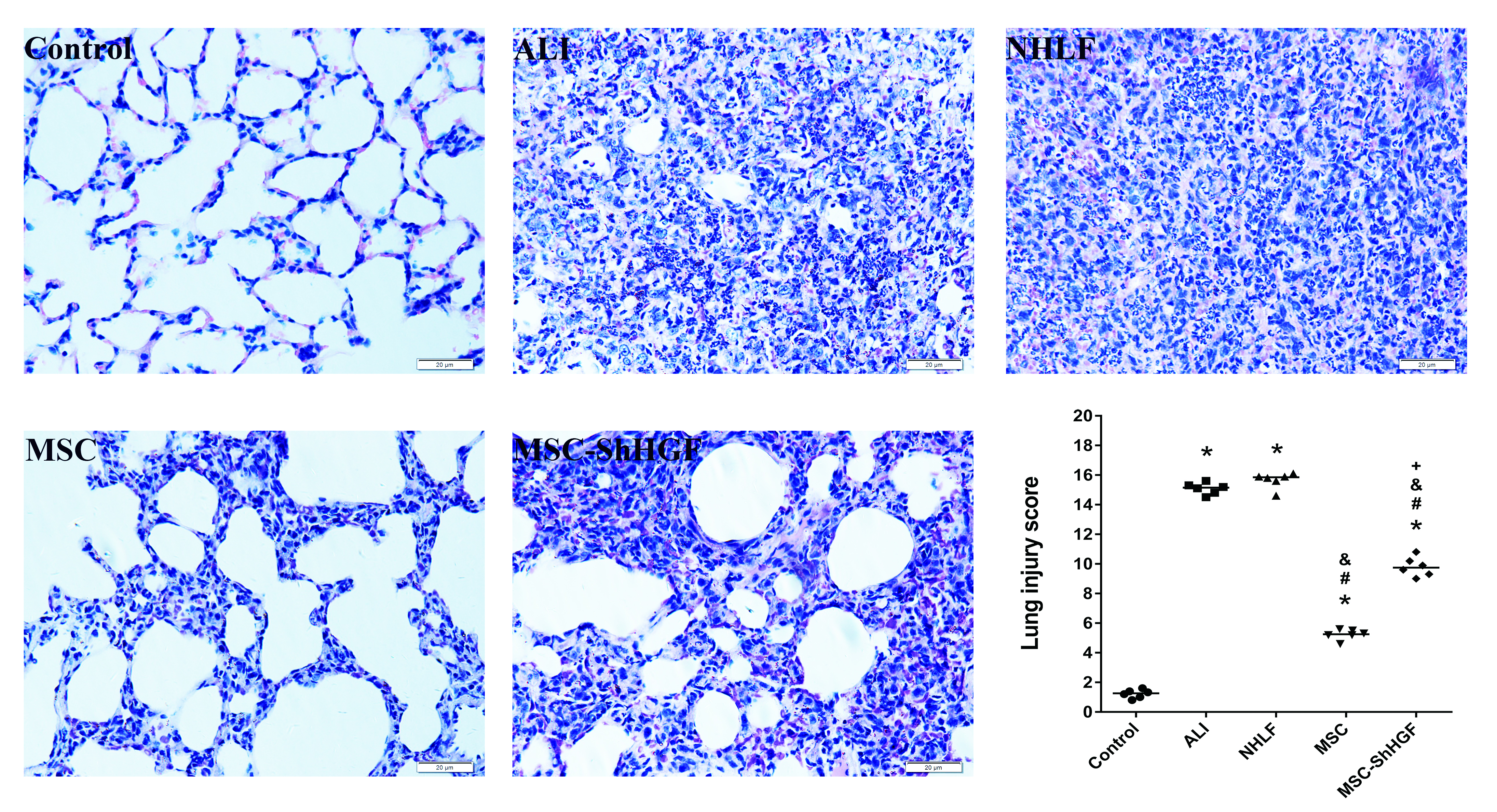


**Figure S4. Histological evaluation of the therapeutic potential of MSC or MSC-ShHGF in ALI rats at 24 hours.** The picture above shows the H&E staining images (×400) of lung sections and a quantitative analysis of the lung injury scores (LIS) in each group at 24 hours. The H&E staining images showed inflammatory infiltrates, interalveolar septal thickening, and interstitial and alveolar edema in each group at 24 hours. The lung injury score was 1.22 in the control group, 15.08 in the ALI group, 15.60 in the NHLF group, 5.23 in the MSC group and 9.80 in the MSC-ShHGF group at 24 hours. The administration of MSC and MSC-ShHGF significantly attenuated lung injury at 24 hours. However, the lung injury score in the MSC-ShHGF group was significantly higher than that in the MSC. There was no significant difference in lung injury score between the ALI and NHLF groups. (n=6; ^＊^*p*<0.05 vs. the control group, ^＃^*p*<0.05 vs. the ALI group, ^&^*p*<0.05 vs. the NHLF group, ^+^*p*<0.05 vs. the MSC group).
