# Supplementary material for: The hepatocyte growth factor-expressing character is required for mesenchymal stem cells to protect the lung injured by lipopolysaccharide in vivo
Source: Stem Cell Res Ther. 2016 Apr 29;7:66. doi: 10.1186/s13287-016-0320-5 (PMC4850641; doi:10.1186/s13287-016-0320-5)
Supplement: Additional file 3: — Equation for samlpe size calculation. (DOCX 13.5 kb) [file 13287_2016_320_MOESM3_ESM.docx]

**Equation for samlpe size calculation**

N=2*[(α+β)^2^ *σ^2^]/(μ_1_-μ_2_)^2^

N is the sample size in each group.

αis the p value.

βis type Πerror.

σis individual variance or standard deviation.

μ_1_-μ_2_ is the minimal relevant difference between the studied groups.

**Table S1 Multipliers for conventional values of αandβ**

|  | α | |  | β | | | |
| --- | --- | --- | --- | --- | --- | --- | --- |
|  | 0.05 | 0.01 |  | 0.2 | 0.10 | 0.05 | 0.01 |
| Multipliers | 1.96 | 2.58 |  | 0.842 | 1.28 | 1.64 | 2.33 |
